# Current Evidence on and Clinical Implications of Vitamin D Levels in Pain and Functional Management of Knee Osteoarthritis: A Systematic Review

**DOI:** 10.3390/clinpract14050158

**Published:** 2024-09-26

**Authors:** Bianca Georgescu, Adelina Elena Cristea, Doinița Oprea, Andreea Alexandra Lupu, Liliana-Elena Stanciu, Erdin Borgazi, Bogdan Marian Caraban, Viorela Mihaela Ciortea, Laszlo Irsay, Mădălina Gabriela Iliescu

**Affiliations:** 1 Medical Doctoral School, Faculty of Medicine, Ovidius University of Constanta, 1 University Alley, Campus—Corp B, 900470 Constanta, Romania; neagu.bianca96@gmail.com (B.G.); adelinaelena.aeu@gmail.com (A.E.C.); 2 Department of Physical Medicine and Rehabilitation, Faculty of Medicine, Ovidius University of Constanta, 1 University Alley, Campus—Corp B, 900470 Constanta, Romania; doinita.oprea@365.univ-ovidius.ro (D.O.); liliana.stanciu@365.univ-ovidius.ro (L.-E.S.); 3 Department of Orthopedics, Faculty of Medicine, Ovidius University of Constanta, 1 University Alley, Campus—Corp B, 900470 Constanta, Romania; erdin.borgazi@365.univ-ovidius.ro; 4 Department of Plastic Surgery, Faculty of Medicine, Ovidius University of Constanta, 1 University Alley, Campus—Corp B, 900470 Constanta, Romania; bogdan.caraban@365.univ-ovidius.ro; 5 Department of Rehabilitation Medicine, University of Medicine and Pharmacy “Iuliu Hatieganu”, 8 Victor Babes Street, 400012 Cluj-Napoca, Romania; viorela.ciortea@yahoo.com (V.M.C.); irsaylaszlo@gmail.com (L.I.)

**Keywords:** knee osteoarthritis, vitamin D deficiency, medical rehabilitation

## Abstract

Background: Osteoarthritis is a common chronic disease that affects quality of life and increases public health costs. Knee osteoarthritis is a frequent form, marked by joint degeneration, pain, stiffness, and functional restrictions. Factors such as age, genetics, joint injuries, obesity, and vitamin D deficiency can affect knee osteoarthritis progression. While the exact link between vitamin D and osteoarthritis is still being studied, recent research indicates that low vitamin D levels might influence the articular cartilage’s structure and function, potentially accelerating osteoarthritis. This review aims to analyze the last decade of research on vitamin D’s role in osteoarthritis. Methods: A systematic review of the literature was conducted in accordance with the PRISMA guidelines (Preferred Reporting Items for Systematic Reviews and Meta-Analyses). Relevant studies from the last ten years were included to evaluate the association between vitamin D levels and knee osteoarthritis. The inclusion criteria were studies examining the role of vitamin D in cartilage health and osteoarthritis progression and the potential clinical implications for disease management. Results: This review identified a variety of studies exploring the connection between vitamin D and osteoarthritis, with mixed findings. Conclusions: The relationship between vitamin D and knee osteoarthritis remains inconclusive, highlighting the need for further research. An updated evaluation of the literature is crucial for osteoarthritis management strategies and to potentially include vitamin D supplementation in therapeutic protocols.

## 1. Introduction

Osteoarthritis (OA) represents a leading cause of disability among older adults worldwide and is a burden on healthcare systems due to necessitating medical care, surgical interventions such as replacement surgery, and many other management strategies. OA poses a significant public health challenge due to its prevalence and its impact on individuals’ quality of life [1]. As life expectancy is increasing and OA is more prevalent in older people, the global prevalence of OA is expected to increase [2]. Current research indicates a lack of disease-modifying drugs for OA, highlighting the critical need for alternative strategies to prevent the onset and progression of this condition [3].

Osteoarthritis of the knee (KOA) is one of the most prevalent types of OA and is characterized by progressive degeneration of the knee joint structures, leading to pain, stiffness, and functional impairment [4]. KOA affects all components of the knee joint, including the joint capsule, cartilage, synovial membrane, subchondral bone, ligaments, periarticular muscles, menisci, and infrapatellar fat pad [5,6]. The articular cartilage matrix and the entire joint are affected by fibrosis, ulceration, loss of cartilage, hyperemia of the synovial membrane, and sclerosis of the subchondral bone [6]. Initial changes appear in the structures of collagen and proteoglycans, and they determine the degree of meniscal damage and erosion in the articular cartilage [7]. The meniscus suffers rupture of the fibrocartilage, which is a reorganization of this structure due to a loss of substance and unorganized collagen fibers [8]. The subchondral bone is affected due to a hypomineralization process, the appearance of osteophytes, microfractures, and sclerosis. The subchondral bone undergoes a remodeling process [3,6,9]. Ligaments are also influenced by KOA due to fiber reorganization or cystic changes, vascular proliferation, and myxoid degeneration [10]. KOA also leads to weakness of the periarticular muscles (quadriceps) and gastrocnemius, which appears due to a reduction in the extension of the knee [11]. The infrapatellar fat pad secretes a varying number of cytokines and adipokines, which leads to an increase in inflammation and a characteristic change in its structure—fibrosis—which is considered typical for KOA [12]. Regarding the implications of biomarkers that are involved in joint tissue turnover, inflammation, and cartilage degradation, recent research discovered carboxy-terminal cross-linked telopeptides of type II collagen (CTX-II), which reflects cartilage breakdown, and cartilage oligomeric matrix protein (COMP), which is associated with cartilage and joint damage. Inflammatory cytokines and matrix metalloproteinases (MMPs) also play a role in OA progression. These biomarkers can help with diagnosing OA early [13,14].

KOA can be influenced by a variety of factors, including age, genetics, joint injuries, and body mass index [5]. Women, especially in the postmenopausal stage of life, are at a higher risk of developing KOA due to hormonal changes, especially the decrease in estrogen. Woman may experience more severe symptoms than men due to their different biomechanics [7,15]. Conditions such as obesity, metabolic syndrome, and type 2 diabetes are strongly linked to KOA. These disorders contribute to joint degeneration through low-grade inflammation [15]. Regarding associated comorbidities, hypertension and cardiovascular diseases, combined with metabolic disorders, are reported to worsen joint health, as they cause increases in inflammatory responses and oxidative stress, which accelerates OA progression [7]. The disease often develops gradually over time, and individuals may experience worsening symptoms as the disease progresses [16].

There are numerous therapies for managing KOA: non-pharmacologic therapies such as weight loss, exercise, and patient education, and pharmacologic treatments such as nonsteroidal anti-inflammatory drugs (NSAIDs), which are typically used for pain relief [17]. In terms of diagnostic methods, besides magnetic resonance imaging (MRI) and X-ray imaging, some studies show that we can distinguish healthy patients from OA patients through using vibroacoustic signals, highlighting the potential of non-invasive, sound-based diagnostic methods in early OA detection [18,19].

In addition to the factors mentioned above, there is also ongoing research into the potential impact of vitamin D in the etiopathogenesis of OA [20]. While the exact relationship linking vitamin D and OA is still being studied, it remains crucial to acknowledge that sufficient vitamin D levels are beneficial for overall bone health [21]. Unfortunately, studies show that vitamin D deficiency is widespread and represents a global health issue [22]. A comprehensive analysis of studies from 2000 to 2022 involving 7.9 million participants from 81 countries found that about 15.7% of the included population had vitamin D deficiency (serum 25(OH)D < 30 nmol/L), and 47.9% had vitamin D insufficiency (serum 25(OH)D < 50 nmol/L). This prevalence increases in certain regions and populations, depending on factors such as geographical location, socioeconomic status, age, and sun exposure [23].

Vitamin D plays a crucial role in the human body, not only for its well-known role in calcium and phosphate metabolism, which is essential for bone mineralization [24,25], but also for supporting immune function [26,27], reducing inflammation [28], and promoting overall cellular health [29]. There is significant focus on exploring the relationship between vitamin D and the development of OA, reflecting a growing focus on understanding the potential impact of vitamin D levels on this joint disorder. Recent research suggests that low levels of vitamin D are associated with adverse effects on the articular cartilage’s structure and function, leading to joint pain, limited physical activity, and decreased muscle strength, which result in OA progression [20]. Studies have shown that vitamin D deficiency accelerates the development of age-related KOA, while vitamin D supplementation can improve the articular cartilage structure, reduce joint pain, and enhance functionality and quality of life in OA patients [30,31]. Additionally, vitamin D treatment has been found to attenuate OA pain, inflammation, and cartilage destruction, suggesting its potential therapeutic benefits in managing OA symptoms [32]. A study on patients with KOA demonstrated that maintaining an adequate level of vitamin D over a two-year period results in a decrease in pain [26]. Unfortunately, even if vitamin D has shown effectiveness in improving pain and function in KOA patients, its impact on structural cartilage changes and inflammatory biomarkers remains inconclusive.

This review aims to explore the relationship between vitamin D and KOA, examining the current evidence and potential implications for clinical practice. It evaluates the impact of vitamin D supplementation on various aspects of KOA, focusing on disease progression, symptom management (pain reduction and improved joint function and stiffness, as well as muscle strength), and the effects on the overall health and well-being of patients. Although the association between vitamin D and KOA has been investigated in other reviews [30,33,34], the evidence remains controversial. A systematic review that examines both symptomatic and structural outcomes in KOA found that vitamin D supplementation may have symptomatic benefits but cannot prevent the structural progression of KOA [33]. On the other hand, another systematic review that focused only on symptomatic outcomes found no significant effect on pain, function, and stiffness [34]. Therefore, an updated review of the literature is important for better management of KOA and potential alternative strategies that can prevent or attenuate the course of this disease.

## 2. Materials and Methods

This systematic literature review was conducted in accordance with the “PRISMA” guidelines (Preferred Reporting Items for Systematic Reviews and Meta-Analysis), which are internationally recognized for their standardization of reporting in systematic reviews and meta-analyses [35]. Additionally, the protocol used for this review was filed in PROSPERO (International Prospective Register of Systematic Reviews; protocol registration: CRD42024570935).

To identify the relevant articles for our study, we analyzed the following databases: National Center for Biotechnology Information (NCBI), PubMed, Scopus, Web of Science, and Cochrane. Furthermore, articles were selected through a direct search on Google Scholar. To search the databases, we used the following keywords: “vitamin D” OR “cholecalciferol” OR “25-(OH)D” OR “25-hydroxyvitamin D” AND “knee osteoarthritis” OR “knee pain.” Since vitamin D was used as a search term, the studies identified primarily focused on vitamin D3 (cholecalciferol), but there was a limited number of studies examining vitamin D2 (ergocalciferol). We decided to include these studies as well, because both forms of vitamin D play important roles in the management of KOA. We restricted our research to studies published between January 2014 and May 2024. The language restriction was set to English.

We sorted the articles using the PICO strategy (patient/problem, intervention, comparison, and outcome). Included study populations were patients—adults or elderly—diagnosed with KOA based on the criteria set by the American College of Rheumatology (ACR) or through clinical and/or radiographic assessment. KOA can be diagnosed based on clinical criteria alone (at least three of the following: knee pain, morning stiffness lasting less than 30 min, crepitus, bony tenderness, bony enlargement, no palpable warmth of the knee joint) or a combination of clinical and radiographic criteria (knee pain and at least one of the following: age over 50 years, morning stiffness lasting less than 30 min, crepitus on active motion, and osteophytes seen on radiographs). The ACR classification typically uses a combination of symptoms, physical signs, and radiographic evidence [36,37]. Radiographic changes were evaluated with the Kellgren–Lawrence grading system, which evaluates the presence of space narrowing, osteophyte formation, subchondral sclerosis, and bony deformity [38]. Reasons for exclusion included studies focusing on populations with secondary OA or other types of arthritis (inflammatory arthritis) and studies involving pediatric populations.

This study compared individuals with low vitamin D status receiving vitamin D supplementation to those who did not receive treatment or received a placebo treatment. The primary outcome was an improvement in symptoms related to KOA and functional management (pain, stiffness, or joint function). We included studies that provide information about measurement of knee pain, stiffness, or joint function using validated scales such as the Western Ontario and McMaster Universities Osteoarthritis Index (WOMAC) [39] or Visual Analog Scale (VAS) [40]. Additional outcomes, such as disease progression (assessed via radiographic changes) or quality of life (assessed using validated scales), were analyzed if there were available data, but they were not required for study selection.

We included studies that met the following inclusion criteria: randomized controlled trials (RCTs) evaluating the effects of vitamin D supplementation in any form or dosage for KOA, cohort studies, or case–control or cross-sectional studies investigating the relationship between 25-(OH)D levels and outcomes related to KOA, including the evaluation of pain, joint stiffness, and physical function.

We excluded reviews, editorial articles, and non-human studies. Articles written in languages other than English, those published more than 10 years ago, as well as books, chapters of books, and conference abstracts were also excluded.

We exported all articles to the reference manager ZOTERO [41], where duplicates were removed. The remaining articles were assessed for eligibility. In addition, the references of every article were carefully examined for more original publications.

To evaluate the validity of the included studies, two independent reviewers used the Cochrane Risk of Bias tool—RoB2 or robvis—which is a special web application to help visualize risk of bias assessments conducted during systematic reviews [42]. Four studies were characterized as presenting some concerns, and the other five studies were rated as having a low risk of bias; none of the studies were characterized as having a high risk of bias (Table 1).

The question on which this review was based is as follows: What is the impact of vitamin D supplementation on the progression, symptom management, and overall health and well-being of patients with OA? This research question focuses on understanding the impact of vitamin D status in patients with OA, considering key outcomes determined by the level of vitamin D by measuring serum 25-(OH)D, disease progression (assessed by radiographic imaging), symptom management (as a reduction in pain severity), decrease in stiffness, and improvement in physical function.

## 3. Results

The initial search indicated 812 articles (Table 2): 509 in PubMed, 102 in Scopus, 141 in Web of Science, and 60 in Cochrane.

**Figure 1:**
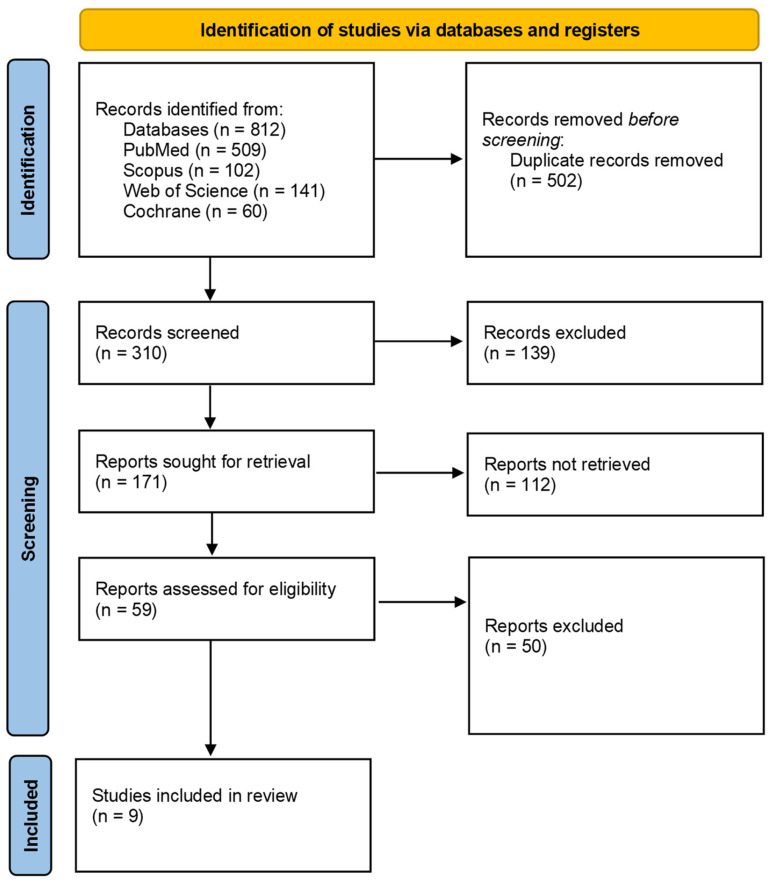
PRISMA flow diagram.

After duplicate removal, 310 articles remained. After screening the titles and abstracts, 139 studies were excluded. From the remaining 171 articles, a selection was made after reading the full text, resulting in 59 articles. Of these, 22 articles had outcomes that were not the subject of our interest, 23 articles were cross-sectional, and 4 were case–control studies, leaving only articles related to vitamin D supplementation in KOA for our analysis (Figure 1).

The resulting articles included in this review (nine) were tabulated in a pre-established table that included the main author’s name, study design, number of patients included, type of vitamin D administered, frequency of administration, and duration of treatment (Table 3 and Table 4).

In a prospective interventional study on 67 patients with KOA who received 50,000 IU oral vitamin D3 (cholecalciferol) weekly for at least 2 months, the author assessed the quadriceps muscle strength in both limbs using a dynamometer and the intensity of knee pain using the VAS and WOMAC pain scales. Serum 25(OH)D reached sufficient levels in almost everyone, except one participant, after supplementation. At the end of the study, participants presented a significant improvement in quadricep muscle strength and a decrease in knee pain [43].

Arden et al. conducted a double-blind, randomized, placebo-controlled trial on 474 participants compared with 237 participants who received 800 IU vitamin D3 (cholecalciferol) daily with 237 receiving a placebo for 3 years. They analyzed the radiographic progression of KOA in the medial compartment of the joint and variations in the WOMAC pain scores, function, and stiffness subscales. They concluded that there is no beneficial effect of vitamin D supplementation on the rate of structural worsening of KOA when assessed by means of changes in joint space width (JSW) or in knee pain, function, and stiffness [44].

In 2016, Jin et al. conducted a study on 413 participants with symptomatic KOA, who were arbitrarily distributed either to a vitamin D supplementation group or to a placebo group. The intervention group was administered monthly oral treatment of vitamin D3 (cholecalciferol)—50,000 IU—for 2 years. There were two primary outcomes of the study: The tibial cartilage volume was evaluated using magnetic resonance imaging (MRI), and knee pain was measured using the Western Ontario and McMaster Universities Osteoarthritis Index (WOMAC) pain scale. The study found no notable difference regarding the volume of tibial cartilage between the vitamin D supplementation and placebo groups over the study period. This finding suggests that vitamin D did not possess a protective effect concerning knee cartilage loss. Regarding knee pain, the results were similar, showing no noticeable difference in the reduction in knee pain between the two groups under study. This indicated no significant improvement in knee pain in the vitamin D group compared with the placebo group [45].

In 2017, Manoy et al. conducted a controlled before–after study on 175 participants with KOA who were administered 40,000 IU vitamin D2 (ergocalciferol) weekly for 6 months. They evaluated knee pain using the WOMAC and VAS scales, muscle strength using a grip strength dynamometer, and physical performance using the following tests: the 4 m gait speed test, Timed Up-and-Go Test (TUGT), Sit-to-Stand Test (STS), and six-minute walk test (6MWT). They also evaluated the quality of life, assessed using the SF-36 questionnaire, and physical activity, assessed using the Physical Activity Questionnaire for Elderly Japanese (PAQ-EJ)—Thai version. The results showed significant improvement in VAS score, SF-12, grip strength, and physical performance after supplementation treatment. They analyzed a series of metabolic risk factors and biochemical markers at baseline and after 6 months. The study found an inverse relationship between 25(OH)D and Il-6 at baseline and that 25(OH)D was negatively correlated with leptin levels after vitamin D supplementation [46]. However, this study presents certain limitations, specifically the lack of a control group.

Another post hoc study by Wang et al. studied the effects of vitamin D3 (cholecalciferol) supplementation regarding knee effusion synovitis volume, measured using MRI. They compared 209 participants who received 50,000 IU vitamin D3 (cholecalciferol) monthly with 204 participants who were a placebo group. In the vitamin D group, the effusion synovitis volume remained stable, but it increased in the placebo group after 24 months [47].

In a post hoc analysis, Zheng et al. used data from the previous RCT to explore additional insights and clarify certain aspects of their primary findings. They measured serum 25(OH)D levels at baseline and follow-up after 3 and 24 months to determine the vitamin D status. They grouped the participants based on their serum vitamin D levels as consistently insufficient (25(OH)D ≤ 50 nmol/L at both follow-up points), fluctuating (25(OH)D > 50 nmol/L at one point), or consistently sufficient (25(OH)D > 50 nmol/L maintained through any follow-up point during the study period). They measured the tibial cartilage volume using MRI at baseline and 24 months and assessed symptomatic outcomes using the WOMAC subscales for pain, function, and stiffness at baseline and month 3, 6, 12, and 24. Participants who sustained sufficient serum vitamin D levels experienced less tibial cartilage volume loss than individuals with insufficient levels did. Regarding the symptomatic outcomes, those with sufficient vitamin D levels reported less knee pain and better physical function compared with those with insufficient levels [48].

Perry et al. carried out a randomized, double-blind, placebo-controlled trial on 50 participants who were randomly grouped into a vitamin D group (24), in which participants received 800 IU of daily of vitamin D3 (cholecalciferol), and a placebo group (26). The primary outcome of the study was to observe whether there were any modifications in synovial tissue volume and bone marrow lesion (BML), assessed by means of MRI. They found no notable difference in the synovial tissue volume or subchondral BML volume between the vitamin D and placebo groups after 24 months [49].

Divjak et al. conducted an open-label clinical trial on 80 patients with primary KOA who were randomly allocated to one of two groups: the vitamin D supplementation group, in which participants received 4000 IU oral vitamin D3 (cholecalciferol) (60) daily for 3 months, or the no-supplementation group (20). They investigated symptomatic outcomes assessed based on WOMAC subscores and VAS and serum levels of certain cytokines using the ELISA method. Their findings suggested improvements in knee pain, stiffness, and function and significant changes in the expression of specific inflammatory mediators in the vitamin D group compared with the placebo group [50]. Unfortunately, the short period of treatment is a limitation of this study.

Another study published last year by Saengsiwaritt et al. researched the effect of vitamin D supplementation on circulating levels of the autophagosome protein marker LC3A, inflammation, and physical performance in patients with KOA. The participants received 40,000 IU of vitamin D2 (ergocalciferol) for 6 months. The serum LC3A level was notably higher in patients with KOA compared with the healthy control group, and after the supplementation, it decreased significantly. In addition, vitamin D2 supplementation for 6 months led to a significant improvement in muscular strength, assessed based on grip strength and knee extension force, and physical ability, assessed using the gait speed test, TUGT, STS, and 6MWT. Furthermore, vitamin D2 (ergocalciferol) supplementation decreased the knee pain score, metabolic biomarkers (LDL and HDL cholesterol), PTH levels, inflammatory markers (hs-CRP and IL-6), and oxidative stress indicators (protein carbonyl) [51].

## 4. Discussion

The impact of vitamin D on treating or preventing OA remains debated. Multiple studies have been published, but most are observational studies, with only a few being controlled trials. OA represents the most common pathology of the musculoskeletal system, especially in elderly patients. Despite current therapeutic interventions that are focused on symptom management, KOA remains one of the most common joint pathologies, with major impacts on patients’ quality of life, as it is characterized by progressive structural joint deterioration. The disease trajectory is heterogeneous, and patients can experience rapid disease progression, which can lead to disability [52]. These impacts underscore the urgent need for novel therapeutic strategies that directly target disease-modifying pathways and prevent structural damage. A study conducted in 2019 regarding the influence of vitamin D on KOA showed that this vitamin influences osteoblastic and chondrocyte cell functions. It is involved in chondrocyte maturation and osteoblastic proliferation and establishes mineralization. It also stimulates proteoglycans and osteocalcin, which are essential for cartilage health [52]. As another study reveals, OA is characterized by subchondral bone hardening, which involves complex bone remodeling processes that affect the bone structure [53]. These findings led to the idea that vitamin D supplements might reduce the degradation of knee cartilage, but the evidence is controversial. Even in our study, we analyzed a study that showed that vitamin D supplementation in patients with KOA did not influence cartilage loss [45], as well as another study that showed that those who took vitamin D did not lose cartilage [48], which shows that it does provide some articular cartilage benefits. This highlights the central aim of our study, which is to rigorously evaluate whether vitamin D supplementation has a measurable impact on the progression of KOA.

There are several studies that reveal that optimal levels of vitamin D in patients with KOA are related to maintaining a good quality of life [54] or that the administration of vitamin D results in a lower degree of disability for these patients [55]. Patients with knee osteoarthritis who have a vitamin D deficiency experience increased pain [56,57,58], reduced mobility, and a lower overall quality of life [56], although there are studies that do not claim that pain is influenced by the low level of vitamin D [59,60,61,62]. Low levels also induce anxiety and influence patients’ participation in social events due to the impact of physical impairments [57,63]. Sufficient vitamin D levels are associated with decreased pain [43,64] and improve depressive symptoms in patients with KOA [64,65]. Upon reviewing the existing literature, which consistently highlights the controversy regarding the effects of vitamin D supplementation on knee pain, the quality of life of patients, or disability in patients with KOA, we further emphasize the need for this study. These findings underline the ambiguity surrounding vitamin D’s role, particularly in terms of clinical outcomes, thus reinforcing the importance of investigating its potential therapeutic impact more thoroughly.

Regarding knee effusion synovitis, there are different conclusions as well: Depending on the studies conducted, no significant improvement was found [49], the volume remained stable [47], or the effusion increased less in patients who took vitamin D [66,67]. Thus, further studies may be needed to ascertain whether there really is an influence on effusion synovitis. Given these mixed results, this review serves as a critical step in addressing the gaps in the current evidence and guiding future clinical interventions.

Serum levels of vitamin D3 have been associated with the severity of KOA in several studies [31,68,69,70,71], in correlation with specific radiological changes [72,73], although some studies concluded that patients who took vitamin D exhibited improved radiological findings [74,75] or that low levels of vitamin D are associated with specific Rx changes in OA [76]. Given the controversial findings in the existing research, with one study in our review in particular showing no significant radiological changes in patients taking vitamin D [44], it has become clear that more detailed investigations are necessary, and this reinforces the objective of our study.

It is known that osteoarthritis is associated with inflammation and high levels of different cytokines, such as IL6, which is also associated with vitamin D3 deficiency [77]. Vitamin D deficiency is linked to increased inflammatory biomarkers [50,51,78,79,80,81] and the severity of the clinical symptoms of knee osteoarthritis [78]. From the articles selected for our study, a clear connection can be established between vitamin D and inflammation in KOA. In particular, a decrease in inflammatory cytokines after vitamin D administration has been shown [50], with other studies demonstrating similar findings.

A loss of muscle mass or muscle weakness can occur due to the development of osteoarthritis through inadequate joint use due to pain, gait, or articular deformities, but osteoarthritis can also occur due to inadequate vitamin D levels. Based on these findings, some studies show that vitamin D is highly associated with increased muscle strength, especially for the quadriceps, which represent the knee stabilizer [43,46,79,80,81,82]. The articles selected for our study align with other research, demonstrating a strong association between vitamin D serum levels and increased muscle strength [43,51]. These findings support the role of vitamin D in improving joint function.

There is no consensus regarding the standard dose of vitamin D that should be administered; the published studies were carried out with doses of between 800 UI and 50,000 UI of vitamin D. There are also studies in which patients were administered vitamin D3 (cholecalciferol) or vitamin D2 (ergocalciferol), although there are only a few on the latter, which revealed almost the same effects as vitamin D3 administration [82]. It is not clear which form of vitamin D is more efficient for osteoarthritis, but the majority of studies conducted used vitamin D3 (cholecalciferol). Regarding the metabolism of vitamin D, a study from 2019 revealed that vitamin D3 (cholecalciferol) administration is more effective in increasing 25(OH)D concentration than vitamin D2 (ergocalciferol) [83]. Given the lack of consensus on a standardized dose of vitamin D or the effective form for clinical benefit, it is essential to conduct detailed studies to establish the optimal form and administration protocol to maximize effects for patients with KOA.

The strengths of this study are as follows: This review focused on articles published within the last 10 years, ensuring that the findings are current and relevant to the field. By selecting the most recent research, this study highlights the contemporary trends and growing understanding of vitamin D’s role in KOA management. This focus on recent studies and the identification of research gaps strengthen the review’s contribution to guiding future clinical trials and therapeutic interventions.

The limitations of this study are as follows: A relatively small number of relevant studies were included, which limits the generalizability of the findings. Additionally, there is no consensus on the standard duration of vitamin D administration, leading to significant variability across studies regarding the dosage, form of vitamin D (D3 cholecalciferaol or D2 ergocalciferol), and treatment period. Not all studies focused on the same outcomes, and their follow-up periods were inconsistent, making it difficult to draw definitive conclusions about the long-term efficacy. These limitations underscore the need for standardized, well-designed trials to better understand the role of vitamin D in the management of KOA.

## 5. Conclusions

Based on the studies reviewed, it is evident that vitamin D administration offers several benefits for patients with KOA. It improves quality of life, reduces pain perception, enhances muscle strength, and lowers inflammation. Additionally, it has shown positive effects on mental health, reducing symptoms of depression and anxiety.

However, further research is needed with lager groups of patients and longer follow-up periods, ideally with standardized vitamin D doses.

As the studies have revealed so far, vitamin D is efficient and has several benefits for KOA, but future studies should prioritize investigating its long-term effects.

## Figures and Tables

**Table 1:** Representation of the reviewers’ gradings of the selected articles.

		Risk of Bias							
		D1	D2	D3	D4	D5	D6	D7	Overall
Study	Heidari et al. [43]	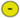	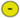	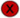	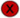	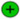	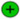	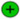	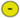
	Arden et al. [44]	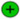	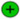	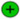	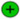	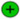	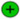	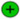	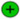
	Jin et al. [45]	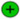	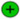	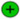	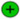	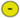	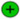	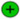	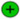
	Manoy et al. [46]	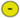	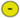	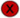	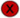	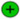	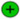	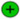	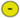
	Wang et al. [47]	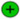	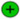	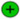	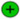	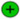	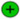	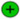	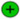
	Zheng et al. [48]	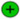	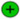	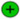	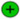	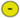	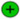	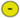	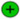
	Perry et al. [49]	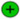	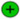	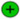	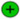	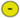	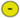	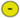	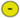
	Divjak et al. [50]	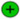	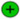	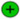	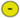	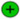	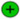	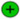	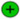
	Saengsiwaritt et al. [51]	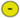	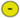	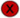	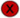	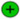	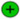	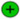	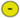
		D1: Random sequence generation D2: Allocation concealment D3: Blinding of participants and personnel D4: Blinding of outcome assessment D5: Incomplete outcome data D6: Selective reporting D7: Other source of bias						Judgement: 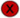 High 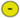 Unclear 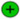 Low	

**Table 2:** Combinations of keywords used to search international databases.

	PubMed	Scopus	Web of Science	Cochrane	Total
Vitamin D AND knee osteoarthritis	193	76	109	54	432
Vitamin D AND knee pain	129	20	22	6	177
Cholecalciferol AND knee osteoarthritis	25	0	0	0	25
Cholecalciferol AND knee pain	20	0	0	0	20
25-(OH)D AND knee osteoarthritis	37	0	3	0	40
25-(OH)D AND knee pain	18	0	0	0	18
25-Hydroxyvitamin D AND knee osteoarthritis	56	3	3	0	62
25-Hydroxyvitamin D AND knee pain	31	3	4	0	38
TOTAL	509	102	141	60	812

**Table 3:** The research articles included in our review.

Authors	Study Design	No. of Patients	Gender	Age (Years)	Dosage	Type of Vitamin	Timing of Treatment	Treatment Duration
Heidari et al. [43]	Prospective double-blind, placebo-controlled, randomized trial	67	86.5% female	50 ± 6.6	50,000 IU	D3 cholecalciferol	Weekly	2 moths
Arden et al. [44]	Randomized double-blind, placebo-controlled trial	474	61% female	64.0 ± 8.0	800 IU	D3 cholecalciferol	Daily	3 years
Jin et al. [45]	Randomized double-blind, placebo-controlled trial	413	50% female	63.2	50,000 IU	D3 cholecalciferol	Monthly	2 years
Manoy et al. [46]	Controlled before–after study	175	90% female	64.58 ± 0.55	40,000 IU	D2 ergocalciferol	Weekly	6 months
Wang et al. [47]	Post hoc analysis of a randomized double-blind, placebo-controlled trial	413	50% female	63 ± 7	50,000 IU	D3 cholecalfiferol	Monthly	2 years
Zheng et al. [48]	Post hoc analysis of a randomized double-blind, placebo-controlled trial	340	50% female	63.2	50,000 IU	D3 cholecalciferol	Monthly	2 years
Perry et al. [49]	Randomized double-blind, placebo-controlled trial	50	74% female	63.3	800 IU	D3 cholecalciferol	Daily	2 years
Divjak et al. [50]	Open-label clinical trial	80	58% female	57.1	4000 IU	D3 cholecalciferol	Daily	3 months
Saengsiwaritt et al. [51]	Prospective single-site, single-arm, nonrandomized interventional trial	175	90.3% female	65	40,000 IU	D2 ergocalciferol	Weekly	6 months

**Table 4:** Articles on the effectiveness of vitamin D treatment for KOA.

Authors	Outcomes Measured	Results after Treatment
Heidari et al. (2014) [43]	- 25-Hydroxyvitamin D level - Knee pain: VAS, WOMAC pain scale - QMS: dynamometer	- 25-Hydroxyvitamin D level increased - WOMAC and VAS decreased - QMS increased
Arden et al. (2016) [44]	- Knee pain: VAS, WOMAC - JSN (X-rays) - 25-Hydroxyvitamin D level	- WOMAC pain decreased in vitamin D group and increased in placebo group - WOMAC stiffness decreased in both groups and WOMAC function increased in both groups - No significant change in JSN - 25-Hydroxyvitamin D level increased in vitamin D group and decreased in placebo group
Jin et al. (2016) [45]	- WOMAC pain score - Tibial cartilage volume (MRI) - Cartilage defects and BML (MRI) - 25-Hydroxyvitamin D level	- No significant differences in WOMAC pain score - No significant differences in change in tibial cartilage volume and BML - 25-Hydroxyvitamin D level increased more in the vitamin D group
Manoy et al. (2017) [46]	- WOMAC and VAS - Quality of life (SF-12) - Physical activity (PAQ-EJ) - Muscle strength (dynamometer) - Physical performance (4 m gait speed test, TUGT, STS, 6MWT); - 25-Hydroxyvitamin D level	- No significant change in WOMAC; VAS decreased - SF-12 improved significantly - No significant change in PAQ-EJ score - No significant improvement in knee extension force - Gait speed, TUGT, STS, and 6MWT improved - 25-Hydroxyvitamin D level increased significantly
Wang et al. (2017) [47]	- Volume of knee effusion synovitis (MRI) - 25-Hydroxyvitamin D level	- Clinical improvements in effusion synovitis volume - Serum 25-hydroxyvitamin D level increased in vitamin D group
Zheng et al. (2017) [48]	- WOMAC score - Structural changes in knee (MRI)	- No differences in WOMAC pain and stiffness - Significant differences in WOMAC physical function - Less loss of total tibial cartilage volume in participants with consistently sufficient vitamin D compared with participants with consistently insufficient vitamin D [33]
Perry et al. (2019) [49]	- STV and BML - 25-Hydroxyvitamin D level	- No significant changes in STV and subchondral BML - Serum vitamin D3 levels increased significantly in the vitamin D group and decreased significantly in placebo group
Divjak et al. (2023) [50]	- WOMAC and VAS - Measurement of cytokine levels - 25-Hydroxyvitamin D level	- In the vitamin D supplementation group, the VAS and WOMAC pain score decreased; the WOMAC pain score increased in the no-supplementation group; the WOMAC stiffness score increased in the vitamin D group and decreased in the no-supplementation group; the WOMAC function score decreased in the vitamin D group and decreased in the no-supplementation group; VAS decreased in the vitamin D group and in the no-supplementation group - IL-1β, IL-23, and IL-33 increased; TNF-α, IL-13, and IL-17 decreased - 25-Hydroxivitamin D level increased in the vitamin D supplementation group
Saengsiwaritt et al. (2023) [51]	- VAS and WOMAC - Muscle strength (dynamometer) - Physical performance - 25-Hydroxyvitamin D level	- VAS decreased significantly - Significant improvements in muscle strength (grip strength and knee extension force) - Physical performance increased (gait speed, TUGT, STS, and 6MWT) - 25-Hydroxyvitamin D level increased

Abbreviation: VAS: Visual Analog Scale; WOMAC: Western Ontario and McMaster Universities Osteoarthritis Index; QMS: quadriceps muscle strength; JSN: joint space narrowing; BML: bone marrow lesion; SF-12: Short-Form Survey; PAQ-EJ: Physical Activity Questionnaire for Elderly Japanese; TUGT: Timed Up-and-Go Test; STS: Sit-to-Stand Test; 6MWT: 6-min walk test; STV: synovial tissue volume; IL: interleukin; TNF: tumor necrosis factor.
